# Prediction of candidate small non-coding RNAs in *Agrobacterium* by computational analysis

**DOI:** 10.1016/S1674-8301(10)60006-1

**Published:** 2010-01

**Authors:** Tingting Zhao, Ren Zhang, Mingbo Wang

**Affiliations:** a The Laboratory Center for Basic Medical Sciences, Nanjing Medical University, Nanjing 210029, Jiangsu Province, China; b Department of Biological Sciences, University of Wollongong, Wollongong, NSW 2522, Australia; c CSIRO Plant Industry, Canberra, ACT 2602, Australia

**Keywords:** small non-coding RNAs, small RNAs, *Agrobacterium tumefaciens* genome, solexa sequencing technology

## Abstract

Small non-coding RNAs with important regulatory roles are not confined to eukaryotes. Recent work has uncovered a growing number of bacterial small RNAs (sRNAs), some of which have been shown to regulate critical cellular processes. Computational approaches, in combination with molecular experiments, have played an important role in the identification of these sRNAs. At present, there is no information on the presence of small non-coding RNAs and their genes in the *Agrobacterium tumefaciens* genome. To identify potential sRNAs in this important bacterium, deep sequencing of the short RNA populations isolated from *Agrobacterium tumefaciens* C58 was carried out. From a data set of more than 10,000 short sequences, 16 candidate sRNAs have been tentatively identified based on computational analysis. All of these candidates can form stem-loop structures by RNA folding predictions and the majority of the secondary structures are rich in GC base pairs. Some are followed by a short stretch of U residues, indicative of a rho-independent transcription terminator, whereas some of the short RNAs are found in the stem region of the hairpin, indicative of eukaryotic-like sRNAs. Experimental strategies will need to be used to verify these candidates. The study of an expanded list of candidate sRNAs in *Agrobacterium* will allow a more complete understanding of the range of roles played by regulatory RNAs in prokaryotes.

## INTRODUCTION

Small non-coding RNAs are recognized as important regulators in eukaryotes and prokaryotes [1],[2]. In bacteria, sRNAs are generally untranslated, and range from 50 to 250 nucleotides in length[2]. Bacterial sRNAs discovered so far can be broadly categorized into two major classes, based on their mode of action[3]. One class consists of sRNAs that act by interacting with RNA-binding proteins to modify the activity of the protein[3]. However, the majority of known sRNAs participate in post-transcriptional regulation by base-pairing with the target mRNA, changing the translation or stability of the mRNA. In many cases, this interaction is mediated by the RNA chaperone protein Hfq[1],[3].

Recent work, often combining experimental and computational approaches, has led to a dramatic increase in the discovery of bacterial sRNAs[3]. The first reported genome-wide searches of sRNAs in *Escherichia coli(E. coli)* started from the observation that the identified sRNAs resided in intergenic regions and were generally conserved in closely related species[1]. Identification of conserved regions outside of protein-coding genes[4], combined with conservation of sequence characteristics expected of non-coding RNAs (stem-loops)[5], has been used successfully to identify them. In other searches, intergenic regions were scanned for promoters and the characteristic DNA sequence and structure of a rho-independent terminator of transcription[6]. Most previous studies employed predominantly computational approaches to predict sRNA genes[7]. These screens were primarily based on searches for sequence conservation among closely related bacteria or searches for promoter and terminator sequences in intergenic regions[4]–[6],[8]. The expression of many of the predicted sRNAs was confirmed by northern analysis of total RNA isolated under a set number of growth conditions[7].

*Agrobacterium* is well known for its natural capability of trans-kingdom DNA transfer[9]. Although a large body of literature has been accumulated from research on this important bacterium, there is currently no information about the presence of small non-coding RNAs and their genes in the *Agrobacterium* genome. The aim of this study was therefore to identify potential Agrobacterial sRNAs using computer-based methods from a short RNA sequence data set recently obtained by a high-throughput sequencing technology.

## MATERIALS AND METHODS

### Short RNA sequences obtained

A set of approximately four million short RNA sequences was provided by Dr Mingbo Wang (CSIRO Plant Industry, Canberra). They were obtained by high-throughput deep sequencing, using the Solexa sequencing technology (Illumina Company, USA) of total RNAs extracted from the plant *Arabidopsis* and *Agrobacterium tumefaciens* strain C58 grown in the presence of acetosyringone. The RNA samples from the two species had been mixed together in a 10:1 ratio prior to sequencing to reduce the cost. Each sequence in the data set was up to 36 bp in length, including the adaptor used in the Solexa sequencing technology (http://www.illumina.com).

### Data sorting and species origin search

Since the millions of short RNA sequence reads were derived from two species and each sequence may contain certain length of the adaptor used at the 3′ end, the first tasks were to find those sequences that belong to *Agrobacteruim* and to remove the adaptor sequence. Duplicate sequences were tallied, then excised to form a non-redundant set of sequences. *Agrobacterium tumefaciens* C58 genome sequences were extracted from NCBI (http://ncbi.nlm.nih.gov) with which the sequence data was compared using a program called SOAP (http://soap.genomics.org.cn), designed for efficient gapped and ungapped alignment of short oligonucleotides onto reference sequences. The adaptor sequences were filtered by SOAP, and the remaining short RNAs were aligned to *Agrobacterium* genome sequences. Short RNA sequences were annotated if an adaptor sequence was detected and the insert sequence matched well with *Agrobacterium* gene sequences. The read was converted to complementary sequence if mapped on the reverse strand. The results were filtered to only retain the top one match for each one. Sequences less than 18 nt were ignored and one mismatch was allowed in this study.

The output of SOAP included short RNA sequence and its ID, sequence quality, total number of matches found for a sequence (number of hits), sequence length, strand matched, match name, match start coordinate, number of mismatches and mismatch description. The results generated from SOAP were combined with the information obtained from Illumina-Solexa sequencing (such as the number of reads, match stop coordinate and so on) by a program designed by Mr. Andrew Spriggs of CSIRO Plant Industry to create a table that involves all of the datasets.

### Computational searches of candidate sRNAs

To identify candidates in our investigations, we took the computational approach that was based on sequence conservation and prediction of secondary structures in RNA folding.

#### Selection of intergenic regions

The short RNA sequences residing in intergenic regions of the *Agrobacterium* genome were identified based on the gene annotations through GenBank accession number of their matched names from NCBI. A coding-region (CDS) was defined as a genomic region that contains an open reading frame (ORF) on either of the two strands, whereas an intergenic region was defined as the one not overlapping with any annotated CDS, rRNA, tRNA, or miscellaneous RNA feature on either strand. Putative 5′UTR regions, 3′UTR regions, flanking regions, as well as function-unknown regions were also considered in this study. Functional regions include protein-coding genes, rRNA genes, tRNA genes, promoters, terminators, regulatory regions and repeats.

#### Identification of candidate sRNA genes by homology

It was assumed that homologous RNA structures would show a reasonable degree of conservation at the sequence level for a given set of genomes. A file containing all known small non-coding RNA sequences of *E. coli* as well as other bacterial non-coding RNA sequences was downloaded from non-coding RNA database (http://ncrnadb.trna.ibch.poznan.pl/download.html)) and used as a starting point for our homology search. The sequences residing in intergenic regions, putative 5′UTR and 3′UTR regions, flanking regions, as well as functionally unknown regions in *Agrobacterium* were used as queries and compared by BLAST (http://ncrnadb.trna.ibch.poznan.pl/blast.html) to the known small non-coding RNA sequences of these bacterial genomes.

#### Screening candidate sRNAs by RNA folding predictions

Since the length of bacterial sRNAs are usually >49 nt, the adjacent sequences with 70 nt in length from both upstream and downstream regions of selected candidates were added based on the *Agrobacterium* genome data from NCBI. The “extended” short RNA sequences were then used for secondary structure predictions. Each strand of the extended sequence was assimilated to a RNA molecule and folded using the RNAfold web server (http://rna.tbi.univie.ac.at/cgi-bin/RNAfold.cgi) with the program's default settings.

## RESULTS

### Basic data obtained

**Fig 1:**
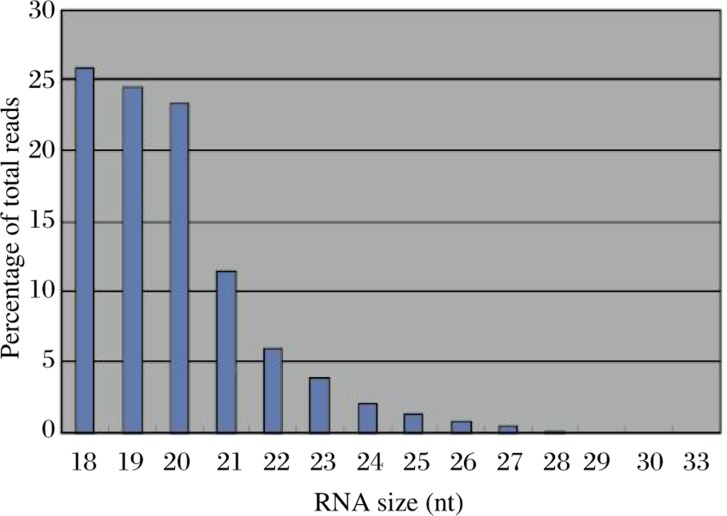
Size distribution of unique RNA sequences obtained from *Agrobacterium*

After searching against the *Agrobacterium* full genome sequence and sorting by the SOAP program, a file of more than 10,000 short RNA sequences was generated. The output of the file included each short RNA sequence of the soil bacterial origin (based on homology search), its ID, matched gene name, total number of matches found for the sequence (number of hits), strand matched, match start coordinate, match stop coordinate, sequence length, mismatch description and number of sequence reads. After adaptor subtraction, these short RNA sequences were in the size range between 18 nt and 33 nt. They were presumed to be authentic sRNAs or degradation products of long RNAs from normal gene transcripts. Most of the sequences matched with *Agrobacterium* 16S ribosomal RNA gene sequence, presumably due to its high abundance in the genome. The total number of reads were 30 998 in the file and some sequences had very high reads (more than 1 000), indicating their high frequencies in the RNA population. The result on size distribution is summarized in ***Fig. 1***.

In general, the genome of *Agrobacterium* is quite compact. Consistent with this, only 4% of the short RNA sequences resided in intergenic regions, whereas more than 90% were from functional regions, and the other 5% occurred in putative 5′UTR, 3′UTR, and flanking regions, as well as function-unknown regions. Sequences which did not match the functional regions were used for further analysis. In total, 869 identified short RNA sequences reside in intergenic regions, putative 5′UTR and 3′UTR regions, flanking regions, as well as function-unknown regions.

### Identification of candidate sRNA genes by homology

As a starting point for detecting sRNAs in *Agrobacterium*, we considered a number of common properties of the previously identified sRNAs of *E. coli* that might serve as a guide. We define sRNAs as relatively short RNAs that do not function by encoding a complete ORF. Since all known sRNAs are encoded within intergenic regions (defined as regions between ORFs), for the conservation analysis, sequences of potential sRNAs in *Agrobacterium* were used as queries and compared against bacterial non-coding RNA database by BLAST program. However, in this homology search, none of the potential Agrobacterial sRNAs shows any significant sequence similarities.

### Screening candidate sRNAs by RNA folding predictions

Among the 869 identified sRNA sequences, many were overlapped, matching the same genomic regions. For these overlapped RNA sequences, the relatively long ones with relatively high frequencies were selected (about 50) and subjected to RNA folding analysis for secondary structure prediction. Since known bacterial sRNAs generally range from 50 to 250 nucleotides in length, adjacent sequences needed to be added to the selected *Agrobacterium* sRNAs for a RNA structure prediction. Out of the 50 short RNA sequences analyzed, 16 candidates of potential small non-coding RNAs in *Agrobacterium* were tentatively identified, based on their putative secondary structures (***Table 1*** and ***Appendix***). The remaining 34 selected sRNAs did not form significant secondary structures, and were therefore not investigated further. The 16 candidate sRNAs ranged from 148 to 178 nucleotides in length and 7 were from intergenic regions. Two of them showed very high sequence frequencies (1871124 and 1167533, ***Table 1***).

**Table 1:** Candidate sRNAs from *Agrobacterium* predicted based on RNA folding

ID of short RNA sequence	GenBank accession number of matched gene	Matched gene name	Number of hits	Strand	Start of short RNA	End of short RNA	Mismatch	Number of reads	Region	Start of candidate sRNA	End of candidate sRNA	Size of candidate sRNA (nt)
1165572	X82941.1	A.radiobacter rosAR gene	1	+	74	94	A->16G40	1	5′flanking	4	164	161
1076360	AB247620.1	Agrobacterium vitis genes for 16S rRNA	21	+	2166	2184		5	intergenic	2096	2254	159
1101007	U59485.2	Agrobacterium tumefaciens AtrC	1	+	28379	28396		2	intergenic	28309	28466	158
1101886	U59485.2	Agrobacterium tumefaciens AtrC	1	+	27924	27941		11	intergenic	27854	28011	158
1412534	AF010180.1	Agrobacterium tumefaciens plasmid pTiC58 TraI region	1	-	151	171	G->21A3	1	5′UTR	81	241	161
1871124	AB102735.2	Agrobacterium tumefaciens rrnD operon genes for 16S rRNA	3	+	86	104		470	5′flanking	16	175	160
1956060	AY027490.1	Agrobacterium tumefaciens ChvD gene	1	+	2556	2573		4	3′UTR	2486	2643	158
333768	CS573987.1	Sequence 1 from Patent WO2007030432	1	+	6398	6416	T->1A40	25	unknown	6328	6486	159
880854	BD224018.1	Gene switch	2	+	155	174		1	unknown	85	244	160
1167533	X96435.1	A.tumefaciens flaA	3	+	1242	1260		2269	intergenic	1240	1395	156
473095	NC_003064.2	Agrobacterium tumefaciens str. C58 plasmid At	3	-	2483	2501		28	intergenic	2413	2584	172
1010101	AB247613.1	Agrobacterium tumefaciens genes for 16S rRNA	8	+	2265	2282	T->3C40	2	intergenic	2195	2370	176
583983	X69388.1	A.tumefaciens sigA gene for vegetative sigma factor	1	+	588	608		2	5′flanking	518	686	169
345524	Y13942.1	Agrobacterium radiobacter genomic DNA for glycerol trinitrate reductase	1	+	387	404		1	5′flanking	317	494	178
1055788	DJ043962.1	Methods for enhancing abiotic stress tolerance in plants and compositions thereof	2	+	10	27		10	unknown	1	148	148
763581	AF065246.1	Agrobacterium tumefaciens strain Chry5 plasmid pTiChry5 scl pseudogene	1	-	3173	3190		3	intergenic	3103	3260	158

Number of hits: total number of matches found for short RNA sequence

Putative secondary structures of the 16 candidates are shown in **Appendix**. The majority of the structures are rich in GC base pairs (ranging from 50 % to 72 %). Three of them (345524, 473095 and 1010101) are followed by a short stretch of U residues ( > 3 uridines), indicative of a rho-independent transcription terminator, whereas some of the short RNAs are found in the stem region of the hairpin (such as 473095, 583983, 1076360 and 1871124), indicative of eukaryotic-like sRNAs. The differences in GC content could point to the different structural requirements associated with the function of the potential sRNAs. Two of the structures contain extensive duplex regions in which the sequenced short RNA fragments reside, raising the possibility that they may be eukaryotic miRNA-like sRNAs.

## DISCUSSION

### Limitations of this study

In our study, intergenic regions as well as putative 5′UTR and 3′UTR regions were considered. However, potential small non-coding RNAs that reside within annotated regions (such as coding-regions) were not included in our search. This was based on the consideration that all known bacterial sRNAs are encoded at genetic loci other than those of the target genes (*trans*-encoded)[10]. However, all currently known plasmid-borne sRNAs are encoded at the same genetic loci as the target genes (*cis*-encoded) and act as antisense RNAs[11]. The restriction of the computer search to intergenic regions as well as 5′ and 3′ UTR may have excluded the class of *cis*-encoded anisense RNAs that are encoded complementary to their target [2].

The Solexa sequencing technology used in this study is not best suited for looking for *E. coli*-like bacterial sRNAs as it gives only 36 nt sequences in length. Since the sRNAs were size-fractionated by gel electrophoresis before adaptor ligation and sequencing, most sRNAs of longer than 36 nt may have been excluded in the sequenced population. Thus, the number of bacterial sRNAs which can be found in the present study could be well below the real number existing in *Agrobacterium*. However, as *Agrobacterium* interacts closely with host plants, it is possible that it encodes eukaryotic-like sRNAs of 20-30 nt in size. Such sRNAs are likely to be identified in the Solexa sequencing data. Considering this possibility, some of the 16 candidate sRNAs from *Agrobacterium* found in this study could be eukaryotic-like sRNAs rather than *E. coli*-like sRNAs. Further work, such as RNA gel blot analysis, is needed to examine this possibility.

### The limits of sequence homology searching

In this study, we have tried but failed to identify *Agrobacterium* sRNAs through sequence conservation analysis against known bacterial non-coding RNAs. The vast majority of bacterial sRNAs known to date have been identified in *E. coli*. With the exception of a few highly conserved sRNAs such as tmRNA and RnpB, most *E. coli* sRNAs are well conserved only among closely related species such as *Salmonella sp.* and *Yersinia sp.*[12]. Consequently, relatively few putative sRNAs have been identified in other species based solely on primary sequence homology with known *E. coli* sRNAs[13]. This could also account for the failure in our analysis. Furthermore, several recent studies have shown that functional sRNA homologues in different species often lack significant sequence similarity[2]. Directly applying the bioinformatics approaches used in *E. coli* to identify sRNAs in other bacterial species have had only limited success[14]. The principal impediment to applying these approaches to other bacteria is that accurately predicting either promoters or transcription factor binding sites requires reliable species-specific consensus sequences, few of these have been experimentally determined in bacterial species other than *E. coli*[14].

### Secondary structure prediction of candidate sRNAs

One important criterion in predicting *E. coli* sRNA genes is that they should be inspected for strong rho-independent termination signals, defined as GC-rich (> 59 %) stem-loop structures followed by a stretch of > 3 uridines[6]. In this study, putative secondary structure of candidate sRNA 345524 do form stem-loop structure with 62 % GC content and followed by a short stretch of U residues, which could be indicative of *E. coli*-like sRNAs. Among the candidates found in this study, nearly a quarter of the short RNAs are embedded in the stem region of the hairpin (such as candidate sRNAs 473095, 1871124), which resembles the feature of eukaryotic-like sRNAs. In Solexa sequencing, the sequence contained in each of the 36 nt reads most likely comes from the 5′ terminus of a RNA molecule. Interestingly, most of the hairpin-like structures are formed by the sequences downstream of the short RNAs. This may suggest that either the short RNAs are the 5′ terminal region of longer bacterial-like sRNA molecules or they are eukaryotic sRNAs processed by RNaseIII-like enzyme from the hairpin-loop structures.

### Identification of sRNAs based on computational predictions

While the most broadly used approach in eukaryotes has been to create cDNA clones of small transcripts[15], a parallel approach has also been used in bacteria[7]. Cloning and sequencing are the methods of choice for small regulatory RNA identification. By using deep sequencing technologies one can now obtain up to a billion nucleotides, and tens of millions of sRNAs, from a single library[16].

Several characteristics of sRNAs make them difficult to identify by experimental techniques or by straightforward computational approaches[2]. RNA genes have not been annotated during genome sequence analysis due to their lack of defined sequence features[7]. RNA genes are also poor targets for mutation screens due to their small size and because they are resistant to frameshift and nonsense mutations since they do not encode proteins[17]. However, in the past few years, several systematic searches have led to the identification of more than 100 small RNA genes in *E. coli*[12].

The first systematic genome-wide screens employed computational approaches to predict sRNA genes[18]. Rivas *et al*[5] developed an algorithm that relied on conservation of RNA structure elements rather than on primary sequence conservation. A computer program (Intergenic Sequence Inspector (ISI)) designed by Pichon and Felden (2003), which automatically selects candidate intergenic regions and displays sequence and structural signatures of RNA genes, can help in identifying bacterial sRNAs. Overall, hundreds of putative sRNA genes were predicted and are awaiting examination[18].

### Future prospects: target identification of sRNAs in bacteria

sRNAs have now made their impressive debut in a variety of bacteria, and are proving to be important components of many regulatory circuits[19]–[21]. Although methods for finding the sRNAs themselves are available and continue to develop, the next challenge will be to develop equally effective methods for finding the targets of these RNAs[1]. For most purposes, in particular for an understanding of the mechanisms of regulation, as well as other potential functions, primary (true) targets should be identified[22].

## CONCLUSION

The current work serves as a blueprint for the initial prediction of a group of potential novel sRNAs in bacteria. In order to find candidate sRNAs in *Agrobacterium*, a computational approach was used in this study and eventually 16 candidates were predicted. However, we only compiled a list of the candidates that were not yet verified experimentally. Therefore, in future work, these candidates need to be tested and examined using experimental strategies. Also, sequencing technologies that allow for longer sequence reads, such as the 454 technology, may have to be used to search for more Agrobacterial sRNAs. We anticipate that the study of an expanded list of candidate sRNAs in *Agrobacterium* will allow a more complete understanding of the range of roles played by regulatory RNAs in prokaryotes and their interactions with host organisms.

## APPENDIX

**Figure:**
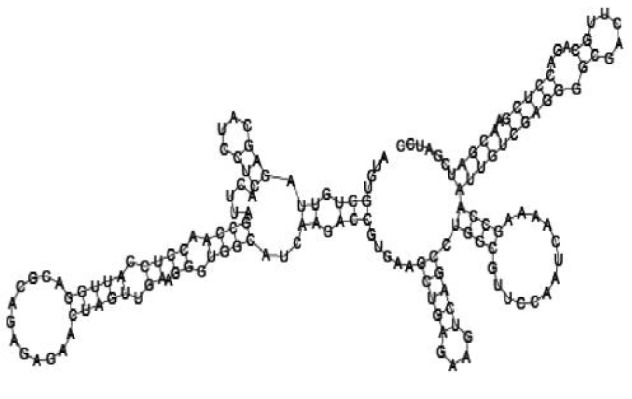
Putative secondary structure of 333768. GC content, 58%

**Figure:**
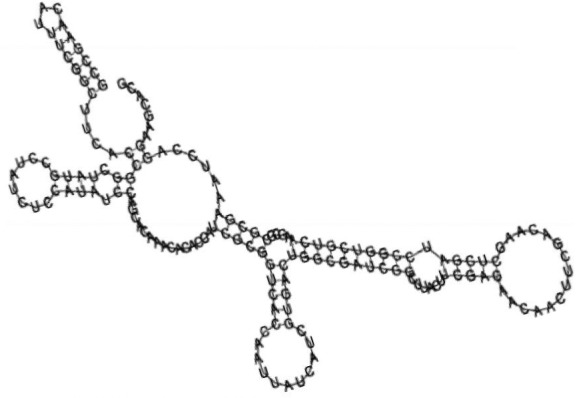
Putative secondary structure of 880854. GC content, 63%

**Figure:**
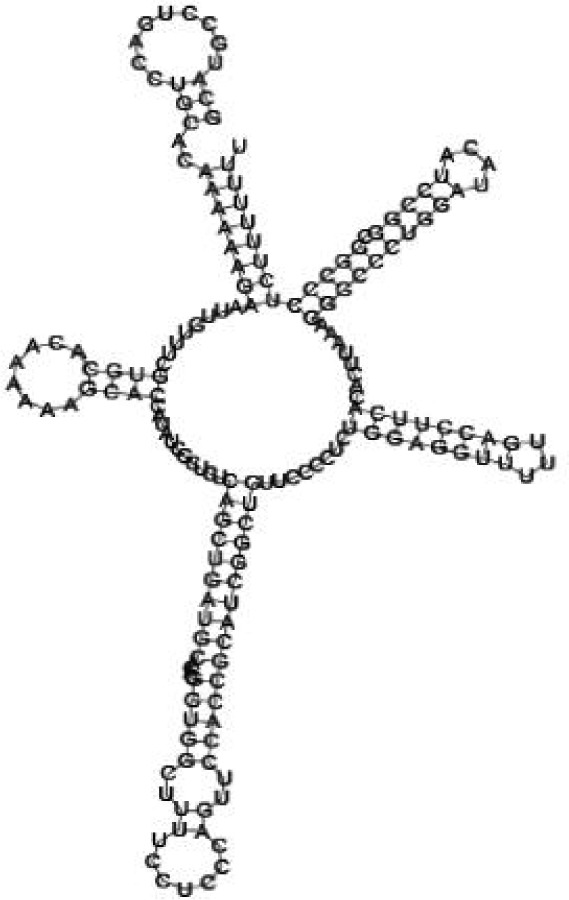
Putative secondary structure of 345524. GC content, 62%

**Figure:**
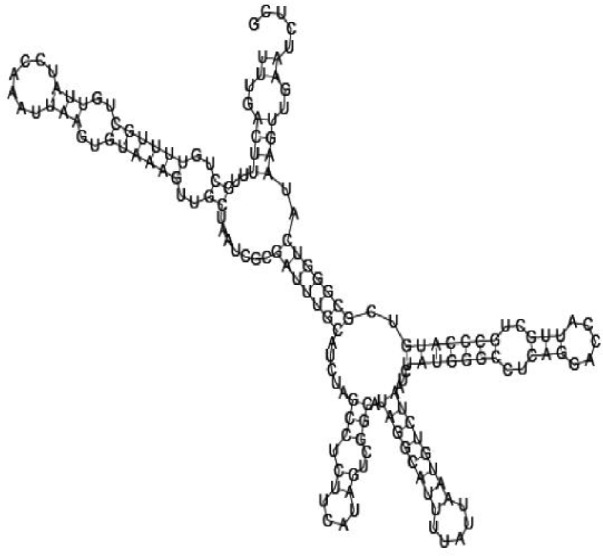
Putative secondary structure of 1101886. GC content, 62%

**Figure:**
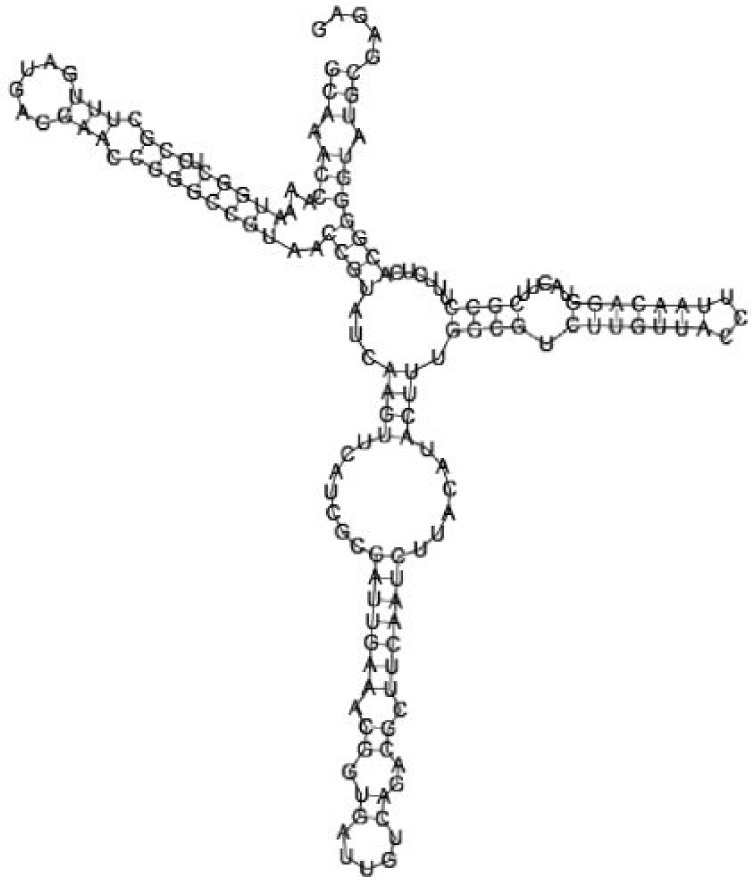
Putative secondary structure of 763581. GC content, 68%

**Figure:**
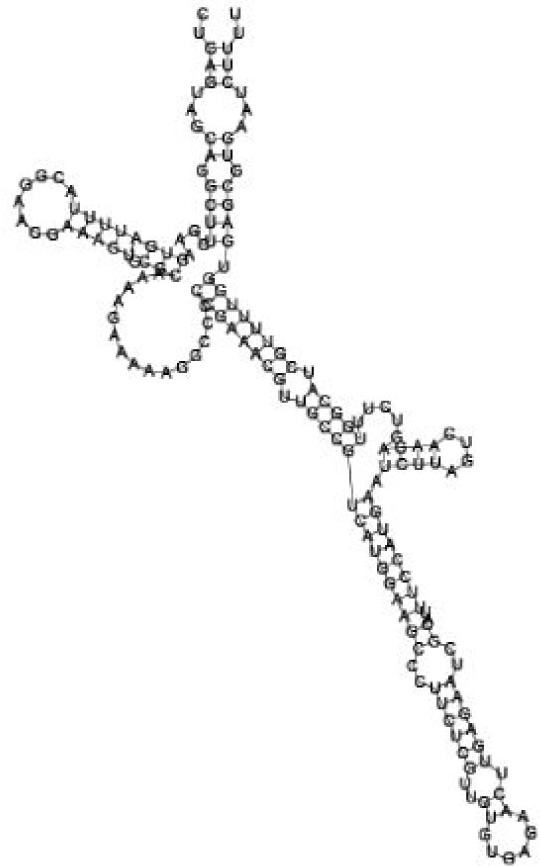
Putative secondary structure of 1165572. GC content, 50%

**Figure:**
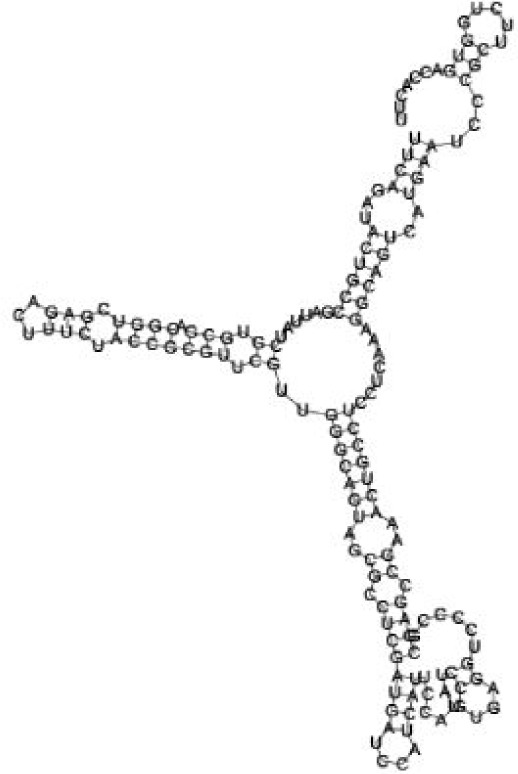
Putative secondary structure of 473095. GC content, 52%

**Figure:**
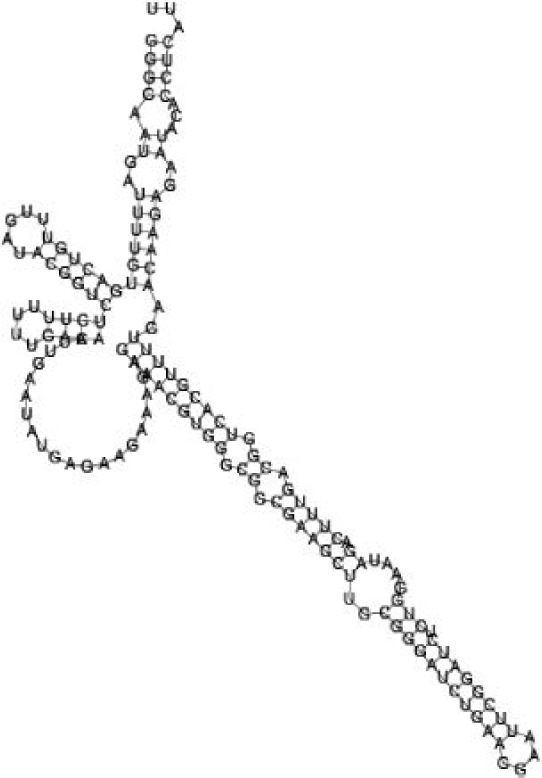
Putative secondary structure of 1871124. GC content, 51%

**Figure:**
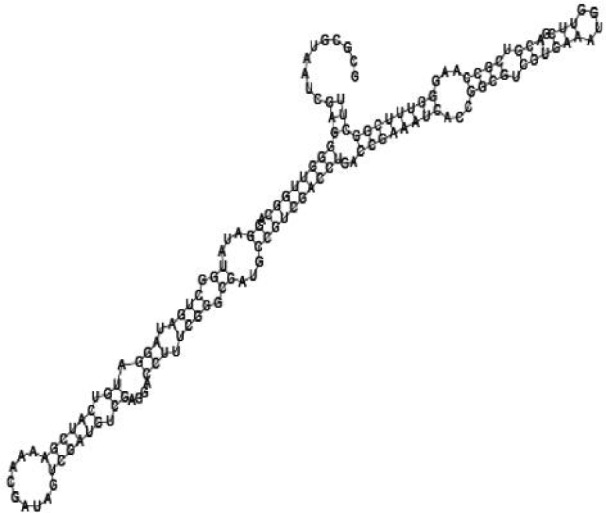
Putative secondary structure of 1055788. GC content, 70%

**Figure:**
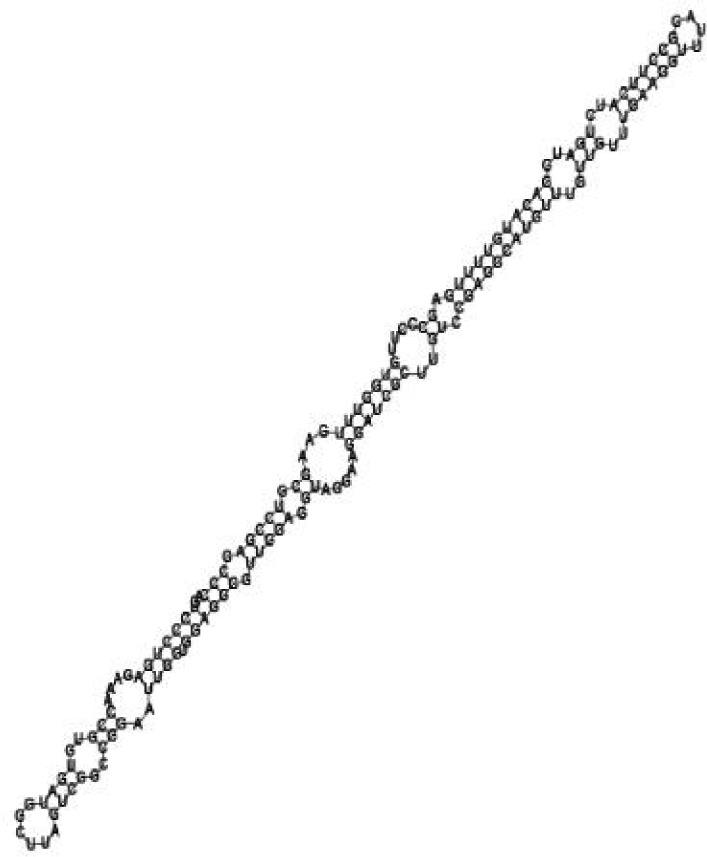
Putative secondary structure of 583983. GC content, 61%

**Figure:**
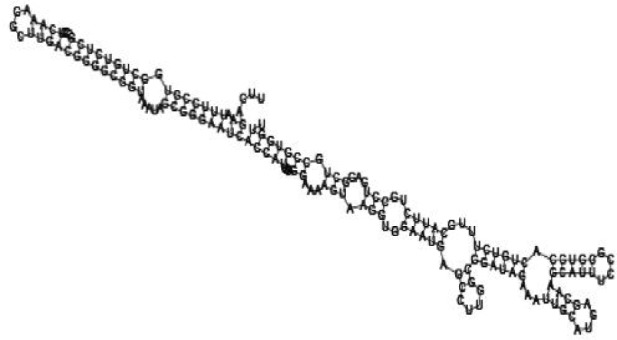
Putative secondary structure of 1010101. GC content, 58%

**Figure:**
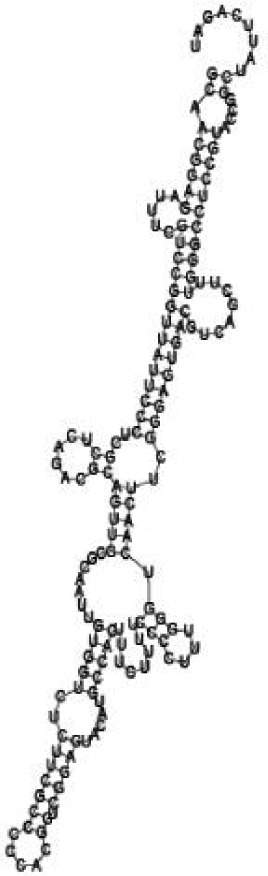
Putative secondary structure of 1076360. GC content, 56%

**Figure:**
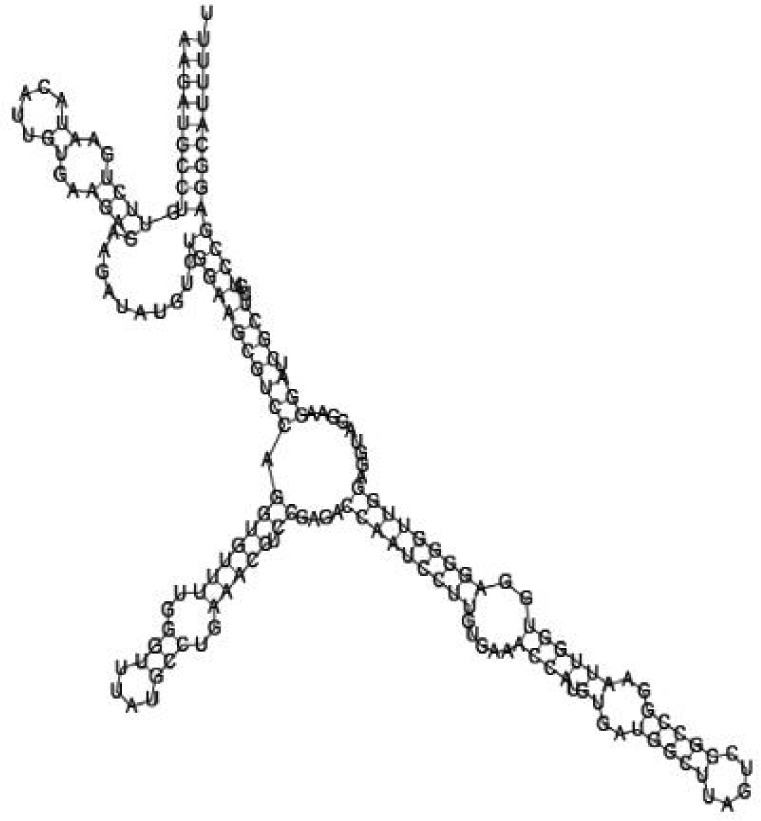
Putative secondary structure of 1101007. GC content, 63%

**Figure:**
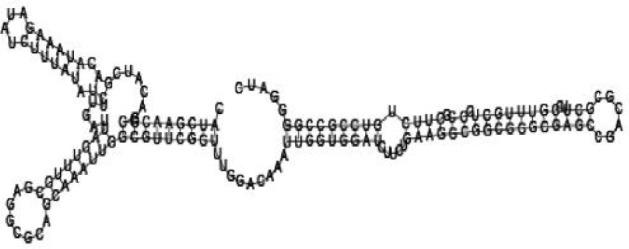
Putative secondary structure of 1956060. GC content, 58%

**Figure:**
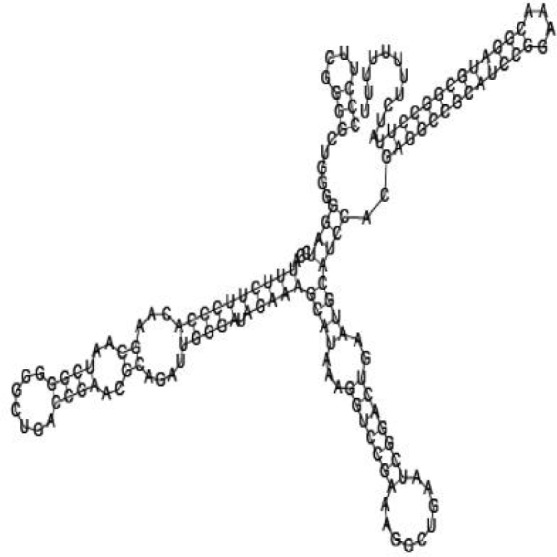
Putative secondary structure of 1167533. GC content, 72%

**Figure:**
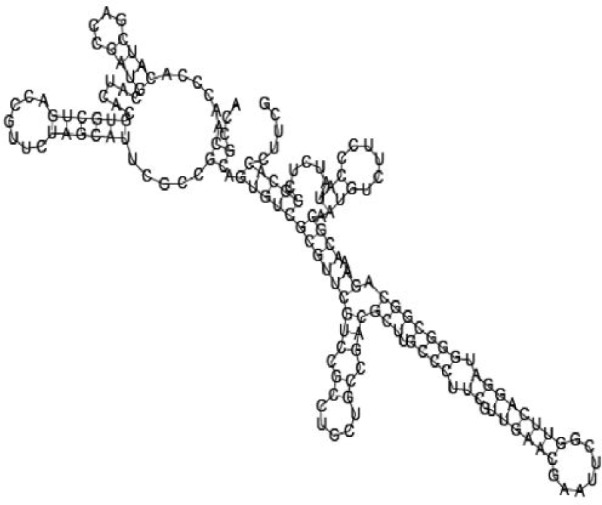
Putative secondary structure of 1412534. GC content, 56%
